# Noncortical coding of biological motion in newborn chicks’ brain

**DOI:** 10.1093/cercor/bhae262

**Published:** 2024-06-25

**Authors:** Elena Lorenzi, Giulia Nadalin, Anastasia Morandi-Raikova, Uwe Mayer, Giorgio Vallortigara

**Affiliations:** CIMeC, University of Trento, piazza della Manifattura 1, Rovereto, TN 30868, Italy; CIMeC, University of Trento, piazza della Manifattura 1, Rovereto, TN 30868, Italy; CIMeC, University of Trento, piazza della Manifattura 1, Rovereto, TN 30868, Italy; CIMeC, University of Trento, piazza della Manifattura 1, Rovereto, TN 30868, Italy; CIMeC, University of Trento, piazza della Manifattura 1, Rovereto, TN 30868, Italy

**Keywords:** noncortical, newborns, brain, *Gallus gallus*, biological motion

## Abstract

Biological motion, the typical movement of vertebrates, is perceptually salient for many animal species. Newly hatched domestic chicks and human newborns show a spontaneous preference for simple biological motion stimuli (point-light displays) at birth prior to any visual learning. Despite evidence of such preference at birth, neural studies performed so far have focused on a specialized neural network involving primarily cortical areas. Here, we presented newly hatched visually naïve domestic chicks to either biological or rigid motion stimuli and measured for the first time their brain activation. Immediate Early Gene (*c-Fos*) expression revealed selective activation in the preoptic area of the hypothalamus and the nucleus taeniae of the amygdala. These results suggest that subpallial/subcortical regions play a crucial role in biological motion perception at hatching, paving the way for future studies on adult animals, including humans.

## Introduction

The movement of living beings is naturally attractive for animals. Johansson (Johansson 1973) first observed that an animation sequence, consisting of just a few strategically positioned points of light on the main joints, suffices to create the impression of an organism engaged in coordinated activity, such as walking. This ability to perceive biological motion has been extensively investigated in several vertebrate species (Herman et al. 1990; Blake 1993; Oram and Perrett 1994; Dittrich et al. 1998; Regolin et al. 2000; Tomonaga 2001; Vallortigara et al. 2005; Vallortigara and Regolin 2006; Parron et al. 2007; MacKinnon et al. 2010; Vangeneugden et al. 2010; Jastorff et al. 2012; Nakayasu and Watanabe 2013; Schluessel et al. 2015; Kovács et al. 2016; Atsumi et al. 2018; Ishikawa et al. 2018; Lorenzi and Vallortigara 2021), it is present at the onset of life (Vallortigara et al. 2005; Simion et al. 2008: 20) and seems to be impaired in neurodevelopmental disorders affecting social cognition such as autism (Klin et al. 2009; Di Giorgio et al. 2016, 2021b).

Brain imaging and electrophysiological studies have identified several brain areas associated with biological motion perception (Pavlova 2012; Lorenzi and Vallortigara 2021). Single-cell recording in awake macaque monkeys (Perrett et al. 1985; Perrett et al. 1990; Oram and Perrett 1994), brain imaging in humans (Grossman et al. 2000; Grèzes et al. 2001; Vaina et al. 2001; Grossman and Blake 2002; Pavlova et al. 2005, 2006; Peuskens et al. 2005; Peelen et al. 2006; Krakowski et al. 2011), and neuropsychological lesion studies (Battelli et al. 2003; Saygin 2007; Gilaie-Dotan et al. 2011) suggest that biological motion perception engages a specialized neural network that differs from processing of other moving stimuli, and that involves portions of the fusiform gyrus (Grossman et al. 2000; Vaina et al. 2001; Peelen et al. 2006), the extra-striate body area (Grossman and Blake 2002; Peelen et al. 2006; Jastorff and Orban 2009), and portions of the parietal (Bonda et al. 1996: 199; Grèzes et al. 2001) and frontal cortices (Saygin et al. 2004) primarily in the right hemisphere. In particular, two brain areas that appear consistently involved in both humans and monkeys are the temporal sulcus (STS; (Oram and Perrett 1994; Jellema et al. 2004; Grossman et al. 2005) and human middle temporal lobe +/V5 (Grèzes et al. 2001; Orban et al. 2003; Peuskens et al. 2005), which is the homolog of monkey middle temporal lobe and its satellites (Vanduffel et al. 2001). Only a small number of studies reported an involvement of subcortical brain regions such as the amygdala and the cerebellum (Sokolov et al. 2014, 2018, 2020; Jack et al. 2017).

This focus on cortical areas is at odds, however, with evidence that perception of biological motion is in fact available at the onset of life in human newborns and in newly hatched chicks (Vallortigara et al. 2005; Simion et al. 2008). Together with face-like appearance (Turati et al. 2002; Rosa-Salva et al. 2010), spontaneous changes in speed (Mayer et al. 2016; Di Giorgio et al. 2021a), self-propulsion (Mascalzoni et al. 2010; Di Giorgio et al. 2016) and other cues for detecting animacy (Rosa-Salva et al. 2018), biological motion is an example of the type of visual signal with which young organisms are equipped from the start of life enabling even newly hatched to identify living from nonliving things.

Evidence suggests that these animacy cues are mediated by sub-cortical (or sub-pallial for animals with a nuclear rather than a laminated cortex or pallium, (Karten 1991) structures, in particular by highly conserved brain areas of the so-called Social Behavior Network (SBN), which comprises the lateral septum, the preoptic area of the hypothalamus, the medial extended amygdala, anterior and ventromedial hypothalamus, and part of the midbrain (Newman 1999; O’Connell and Hofmann 2011; Goodson and Kingsbury 2013; Lorenzi et al. 2017; Mayer et al. 2017a, 2017b).

**Fig. 1:**
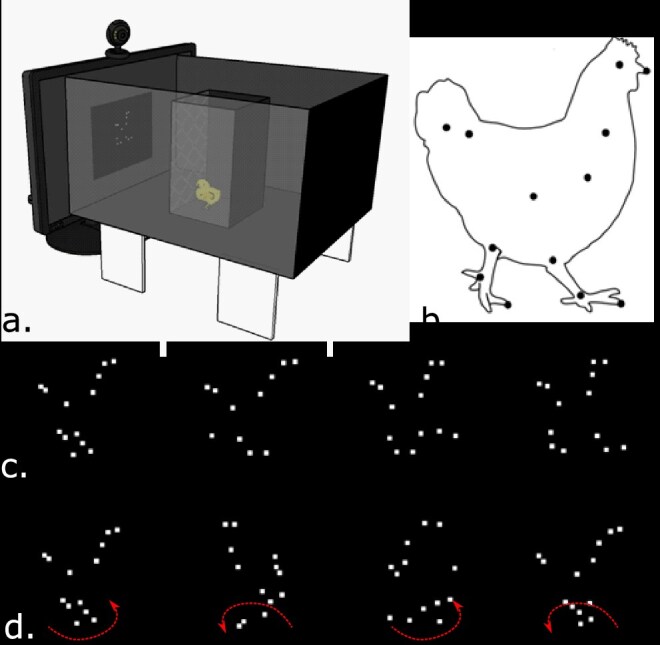
Experimental setup and stimuli. (a) Schematic representation of the experimental apparatus. One of the two long walls is depicted as translucent in order to demonstrate the setup. The chick was located inside the exposure box. The wall of the exposure box, facing the video screen, had a black metal grid that allowed the chick to look at the stimulus. On one side of the apparatus, a monitor was showing the stimulus. A webcam was placed centrally above the video screen to record the chick’s behavior from the front. (b) The walking hen point-light display, modified from (Vallortigara et al. 2005). Black dots indicate the location of each point of light to create the animation. (c) Four frames sampled from the walking hen animation (biological motion stimulus). (d) Four frames sampled from the rotating hen animation (nonbiological motion stimulus). The dotted arrows represent the rotation direction of the single frame.

Here, we investigated for the first-time brain activation as measured by the immediate early gene expression of c-Fos in visually naïve newly hatched chicks. We compared c-Fos expression to biological and nonbiological (rigid; Fig. 1) motion in selected areas of the SBN in order to check whether any selectivity to biological motion could be observed in these sub-cortical (sub-pallial) regions.

## Materials and methods

### Subjects

Subjects were 32 laboratory-hatched (females = 15), domestic chicks (*G. gallus*) of the Ross 308 strain. We tested both male and female chicks because differences in response to biological motion associated with sex have been observed in several species (humans: (Pavlova et al. 2015); monkeys: (Brown et al. 2010); chicks: (Miura and Matsushima 2012; Zanon et al. 2024)). Fertilized eggs were obtained from a local commercial hatchery (Passirano, Brescia (BS)—Italy) and incubated in total darkness within a Marans P140TU-P210TU incubator at a constant temperature of 37.7° and 60% humidity. After hatching, the chicks were kept inside the incubators until they were tested under the same temperature and humidity conditions. All procedures were performed in complete darkness to avoid any visual experience before and after testing.

### Ethics statement

The experiments reported here comply with the current Italian and European Community laws for the ethical treatment of animals and the experimental procedures were licensed by the Ministero della Salute, Dipartimento Alimenti, Nutrizione e Sanità Pubblica Veterinaria (permit number 845/2018).

### Apparatus

The experimental apparatus (see Fig. 1a) was located in a dark room. It consisted of a black wood box (37.3 × 49.5 × 28.3 cm^3^) open on one side to allow the insertion of a video screen (LCD Monitor BenQ XL2410T, 23.6 inches, 1920 × 1080, at least 120 Hz) used for the visual stimulation. In front of the screen at a distance of 16 cm, a black plastic box (14.5 × 14.5 × 23.3 cm^3^) was used to constrain the chick during the visual exposure (15 min). The wall of the exposure box, facing the video screen, presented a black metal grid, enabling the subject to look at the stimulus. The only source of light was the monitor screen that displayed the video stimuli. A webcam was placed at the center of the video screen to record the chick’s behavior from the front. The webcam was connected to a screen in the same room, allowing real-time monitoring of the chick’s behavior during the test. The recorded videos were kept for later behavioral analyses offline.

### Stimuli

The experimental stimuli consisted of point-light animations identical to those used in a previous study to test biological motion preferences in chicks (Miura and Matsushima 2016). Each stimulus was composed by a set of 13 bright white dots, placed at the joints of an invisible hen’s skeleton against a black background (see Fig. 1b). The biological motion stimulus represented the sequence of a walking hen. Twenty-three frames were required to cover an animal’s entire step sequence; then, the digitalized sequence was looped for a total stimulus presentation of 15 min. As a result, the point light display remained in the center of the screen but moved as if the hen was walking rightward on a treadmill (see Fig. 1c; the original moving stimuli used are provided in the Supplementary Materials, Video S1).

The rigid motion stimulus was obtained using a randomly selected single frame (made of 13 points of light) from the walking hen animation sequence, which was moved rigidly around the vertical axis so as to produce the motion of a rotating, rigid hen-like object (see Fig. 1d and Video S2).

The motion stimuli were displayed at a speed of 30 frames/s (54px/s), with a refresh rate of the screen of 144 Hz. Each set of points occupied a window of 237 × 252 pixels in the center of the computer screen at 6 cm from the floor of the apparatus and at a viewing distance of 16 cm from the subject in the exposure box. Stimulus size and speed rate were identical to that used in a previous study investigating the spontaneous preference to approach biological motion patterns in visually naïve newly hatched chicks (Vallortigara et al. 2005). The two animations were presented in a loop for a duration of 15 min, encompassing the entire stimulus exposure.

### Exposure session

Overall, we tested 16 chicks (9 males and 7 females) exposed to the biological motion stimulus and 16 chicks (8 males and 8 females) exposed to the rigid motion stimulus. On posthatching day 1, each subject was taken from the incubator in complete darkness and carefully transported into a dark box to the experimental room. Each animal was individually placed into the exposure box inside the apparatus facing the stimulus (see Fig. 1a). Chicks could freely move inside the box for the whole duration of the exposure (15 min). To ensure that subjects were paying attention to the stimulus, a cut-off was set at 10 min of exposure. If the animal fell asleep before this cut-off, it was excluded from further procedures.

After the exposure, in order to distinguish each subject, they were placed back in the dark incubator in single compartments (cardboard box 11 × 11 × 25 cm^3^) where they remained 60 min until the perfusion. In order to keep the auditory environment as it was before the exposure, single compartments were located in the same incubator with other chicks that were not used for the test.

### Immunohistochemistry

Seventy-five minutes after the start of the exposure, subjects were overdosed with an intramuscular injection of 0.05 mL ketamine/xylazine solution (1,1 Ketamine 10 mg/ml + xylazine 2 mg/ml) per 10 g of body weight. After 5 min, when animals became unresponsive (tested by gently pulling feet and wings), they were blocked on a surgical plate, the thorax was opened, and the heart was exposed. Chicks were perfused transcardially via the left ventricle with cold phosphate-buffered saline (PBS; 0.1 mol, pH = 7.4, 0.9% sodium chloride, 4°C) for 10 min and then fixed with 4% paraformaldehyde (PFA) in PBS for 10 min. The head was then severed from the body, the skin and the eyes were removed, and the skull was transferred to 4% PFA, where it was stored overnight for further postfixation.

On the following day, the skull was fixed on a stereotaxic head holder. A coronal-plane cut was made on the caudal part with a scalpel blade attached to a micromanipulator. In order to ensure that the subsequent sections had the same orientation as in the chick’s brain atlas of Kuenzel and Masson (Kuenzel and Masson 1988), the cut was made at an orientation of 45°. The brain was then carefully removed from the skull under a microscope, the left and the right hemispheres were separated and processed independently. Each hemisphere was covered with a 7% gelatine in PBS containing egg yolk (4.2 g gelatine + 60 mL PBS + 1 egg yolk at 40°C). After cooling, they were postfixated for 48 h in 4% PFA/PBS containing 20% sucrose at 4°C and then transferred to 30% sucrose/0.4%PFA/PBS until they sunk. The hemispheres were frozen individually at −45°C inside a Cryostat (Leica CM1850 UV) for 20 min covered with a 5% ethanol solution.

Four series of 40-μm coronal sections were cut on the Cryostat at −20°C for free-floating staining and collected in PBS. Only the sections of the first series were used for processing. The sections of the other series were kept in a Cryoprotectant solution (30% sucrose/30% ethylene glycol/PBS), used to protect biological tissue from freezing damage, at −25°C as backups.

Between each of the following steps, the sections were washed in PBS (3 × 15 s, 3 × 5 min). Endogenous peroxidase activity was depleted by incubation in 0.3% H2O2/PBS for 20 min. Subsequently, the sections were treated with 3% normal goat serum (S-1000, Vector Laboratories, Burlingame, CA, USA) in PBS for 30 min. The sections were then transferred to the first antibody solution (c-Fos made in rabbit, 1:2000; polyclonal K-25, Santa Cruz Biotechnology, Santa Cruz, CA, USA) containing 0.1% Bovine Serum Albumin (BSA, SP5050, Vector Laboratories) and incubated for 48 h at 4°C on a rotator. The secondary antibody reaction was carried out using a biotinylated anti-rabbit solution (1:200, BA-1000, Vector Laboratories) in PBS for 60 min at room temperature on a rotator. The ABC kit (Vectastain Elite ABC Kit, PK-6100, Vector Laboratories) was used for signal amplification and neurons with concentrated c-Fos protein were visualized with the VIP kit for peroxidase (SK-4600, Vector Laboratories). Sections were then transferred to a 0.9% saline solution and washed individually in distilled water before serial mounting on gelatine-coated slides. Left and right hemispheres were mounted identically oriented on different microscopic slides in order to blind the experimenter while counting at the microscope. The slides were dried at 40°C and counterstained with methyl green (H-3402, Vector Laboratories). They were gradually dehydrated in ethanol (70%, 80%, 90%, 99% ethanol for 3 min each and then placed in Xylene) and coverslipped with Eukitt (FLUKA).

## Analyses

### Brain analyses

Brain sections were examined under a microscope (Zeiss Axio Examiner) at a magnification of 200x and a digital camera (Zeiss AxioCam MRc5). The experimenter was blind to the experimental conditions and to the brain hemisphere identity during the whole procedure of counting the c-Fos-immunoreactive (c-Fos-ir) neurons. Images on the screen (ZEN Imaging software, Zeiss) were adjusted matching the view under the microscope. Successful immunostaining produced dark purple-black stained nuclei. Thus, the nuclei of c-Fos-ir neurons were easily distinguishable from the background and from nonactivated cells, which were stained light green.

For counting, a rectangular enclosure 150 × 250 μm^2^ was placed over the brain regions of interest. This enclosure was placed keeping a minimum distance from a near subdivision and the edge of the brain section. Every activated c-Fos-ir neuron within the same rectangular area was marked on the screen with the “event marker” of the ZEN software, which automatically computed the total number of marked cells.

**Fig. 2:**
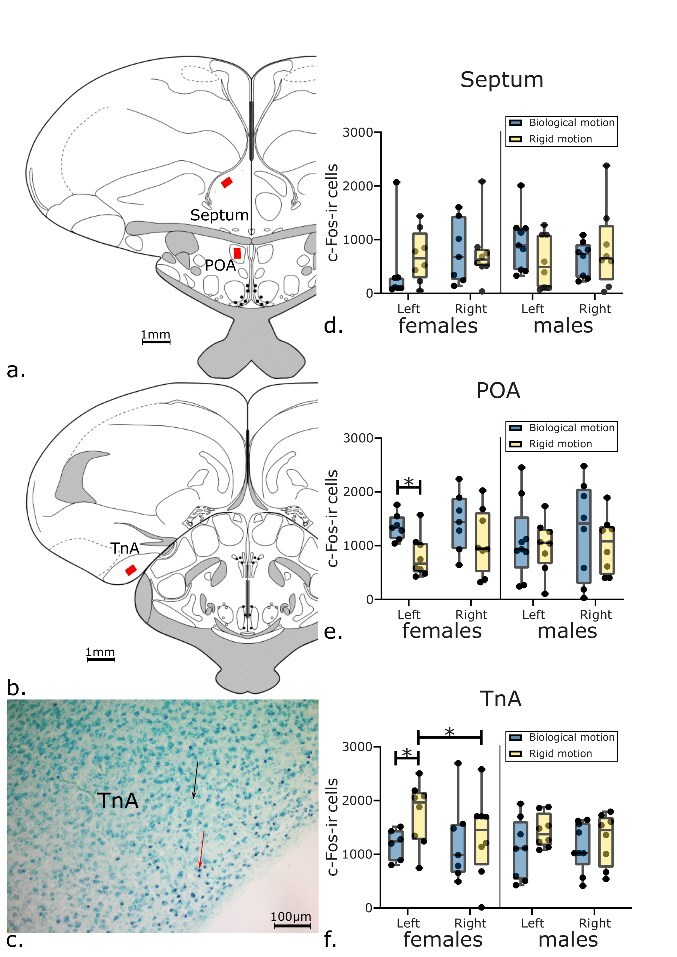
Brain analyses. (a and b) Typical placement of the counting areas (rectangles) in septum, preoptic area (POA) and nucleus taeniae of the amygdala (TnA). The coronal sections’ schematic representations were redrawn from the chicken atlas (Kuenzel and Masson 1988). (c) c-Fos labeled cells in the TnA. Photomicrograph of a coronal section of an experimental chick brain. c-Fos immunoreactive (c-Fos-ir) cell nuclei are stained black ( lower arrow) and are easily discernible from the methyl-green counterstained cells (upper arrow). (d–f) c-Fos-ir cell density estimated in septum, POA and TnA in the two groups of chicks exposed to biological (darker) or rigid motion (lighter). A significantly higher number of c-Fos-ir cells were present in the left TnA of control female chicks compared to the experimental group. The graph plot shows the mean (black square), SEM (box), and SD (whisker), with densities of c-Fos-ir cells per mm^2^ represented on the *y*-axis (^*^ indicates *P* < 0.05). (d) In septum, there was no difference between the two groups. (e) A significantly higher number of c-Fos-ir cells was found in the left POA of experimental female chicks compared to control ones. (f) A significantly higher number of c-Fos-ir cells was found in the left TnA of control female chicks compared to experimental ones. (d–f) No difference was observed in males.

To estimate marked cells density in the septum five sections of each hemisphere were selected by the shape that would correspond to the A8.8 to A8.0 (Kuenzel and Masson 1988) according also to what was done previously in other studies from our lab (Lorenzi et al. 2017; Mayer et al. 2017a, 2017b; see Fig. 2a).

To estimate c-Fos-ir (immunoreactive) cell density within the TnA, four sections of both hemispheres were selected from the region extending from A7.6 to A6.8 in Kuenzel and Masson (Kuenzel and Masson 1988); see Fig. 2b).

As to the preoptic area, counting was done on one section (A8.2; (Kuenzel and Masson 1988) selected from the region where the anterior commissure was apparent. The rectangular enclosure was positioned beneath the anterior commissure, coherently to what was done in previous studies in our lab (Lorenzi et al. 2017; Mayer et al. 2017a; see Fig. 2a).

After the cells were counted, the mean values were calculated per hemisphere for each region and for each individual subdivision of the septum (dorsal, lateral and medial) and cell density was standardized to 1 mm^2^. Counts from the three subdivisions of septum were combined to estimate overall activity in the brain area.

### Behavioral analyses

In order to measure potential behavioral differences between subjects we measured the distance moved and the mean speed at which each subject moved during the exposure session. To this aim, we tracked each subject using the software EthoVision 3.1 (Noldus Information Technology, Leesburg, VA, USA; Noldus et al. 2001).

### Statistical analyses

Before performing any statistical analyses, outliers were identified and removed from the dataset (three measurements). The method employed for outlier detection was Tukey’s, which is based on the data’s interquartile range (IQR, Tukey 1977). This method, that defines outliers as data points that fall below Q1–1.5 ^*^ IQR or above Q3 + 1.5 ^*^ IQR, is effective even with small sample sizes. Indeed, Tukey’s method has shown to reliably detect true outliers, archiving already 50% true positive rate with sample sizes as small as 6 (Coucke et al. 2012).

To detect any difference in the dependent variable “c-Fos density” a repeated measures ANOVA, with two between-subjects factors: “group” (2 levels: biological motion and rigid motion) and “sex” (2 levels: males and females), and two within-subjects factors: “area” (3 levels: septum, TnA and POA) and “hemisphere” (2 levels: left and right) was performed.

For post hoc analyses two-tailed *t*-tests for independent samples were employed to compare c-Fos densities between the two groups and the two sexes. Two-tailed paired *t*-tests were employed to test for lateralization within groups and sexes.

To analyze the two behavioral parameters (“distance” and “velocity”), a multivariate ANOVA with two between-subject factors: “group” (2 levels: biological motion and rigid motion) and “sex” (2 levels: males and females) was performed.

All statistical analyses were performed with the software IBM SPSS Statistics, while the graphs were created with GraphPad Prism 8.

## Results

### Brain activity measurements

The staining process for all 32 brains was successful in detecting c-Fos expression. The use of methyl-green counterstaining resulted in the green staining of nuclei in all cells, while the nuclei of c-Fos-ir neurons appeared black following the immunohistochemical procedure. Consequently, it was easy to distinguish c-Fos-ir neurons from other neurons (see Fig. 2c).

An analysis using repeated measurements ANOVA revealed several significant heterogeneity. There was a significant main effect of “area” (F_(1.890, 47.246)_ = 20.614, *P* < 0.001), indicating that different brain regions exhibited varying levels of c-Fos expression. Additionally, a significant interaction was observed for “area × group” (*F*_(1.890, 47.246)_ = 4.679, *P* = 0.015), “area × hemisphere” (*F*_(2, 50)_ = 4.955, *P* = 0.011), and “area × hemisphere × group × sex” (*F*_(2, 50)_ = 4.243, *P* = 0.020), highlighting complex interactions among these factors. For a comprehensive list of all effects and interactions, see Table S1 in the Supplementary Materials.

Specific differences between groups and sexes were observed (see Fig. 2d–f). In females, a significant difference between the two groups was found in the left POA (*t*_(13)_ = 3.186, *P* = 0.007; Fig. 2e), and in the left TnA (*t*_(10.654)_ = −2.388, *P* = 0.037; Fig. 2f), but not in the left septum (*t*_(13)_ = −0.819, *P* = 0.427; see Fig. 2d).

The left POA of female chicks that were exposed to the biological motion stimulus expressed overall more c-Fos (1352.4 ± 93 cells/mm^2^, mean ± SEM, rounded numbers) than the left POA of female chicks that were exposed to the rigid motion stimulus (806.7 ± 138 cells/mm^2^; see Fig. 2e).

On the other hand, left TnA of female chicks that were exposed to the rigid motion stimulus expressed significantly more c-Fos (1755 ± 207 cells/mm^2^) than the left TnA of females exposed to the biological motion stimulus (1186.3 ± 117 cells/mm^2^; see Fig. 2f).

While no significant effect was present in the left septum of female chicks (rigid motion: 691.8 ± 169 cells/mm^2^; biological motion: 435.1 ± 274 cells/mm^2^; see Fig. 2d).

No significant differences between groups were observed in the right hemisphere in females (right TnA: *t*_(13)_ = −0.263, *P* = 0.797; right POA: *t*_(13)_ = 1.190, *P* = 0.255; right septum: *t*_(13)_ = 0.105, *P* = 0.918; see Table S2 in the Supplementary Materials for all the means ±SEM values).

In male chicks, no significant differences among groups were detected in any of the brain regions examined, including both left and right hemispheres (left TnA: *t*_(14)_ = −1.492, *P* = 0.158; left POA: *t*_(15)_ = 0.318, *P* = 0.755; left septum: *t*_(15)_ = 1.404, *P* = 0.181; right TnA: *t*_(15)_ = −0.611, *P* = 0.551; right POA: *t*_(14)_ = 0.659, *P* = 0.520; right septum: *t*_(14)_ = −0.683, *P* = 0.506; see Table S2 in the Supplementary Materials for all c-Fos cell density values).

To investigate potential sex differences within each experimental group, independent samples *t*-tests were conducted. In both cases, in the group exposed to the biological motion stimulus and in the group exposed to the rigid motion stimulus, no differences between males and females were found in any of the analyzed brain regions in either the left hemisphere (left TnA: *t*_(12)_ = −0.361, *P* = 0.724; left POA: *t*_(14)_ = −0.906, *P* = 0.380; left septum: *t*_(14)_ = 1.621, *P* = 0.127) or the right hemisphere (right TnA: *t*_(14)_ = −0.315, *P* = 0.757; right POA: *t*_(13)_ = −0.437, *P* = 0.670; right septum: *t*_(13)_ = −0.573, *P* = 0.577; see Fig. 2d–f).

Additionally, a paired samples *t*-test was conducted to examine differences between the left and right hemispheres in each brain region for each experimental group and sex. A significant difference was found only in the TnA of females exposed to the rigid motion stimulus (*t*_(7)_ = 2.551, *P* = 0.038), where the left TnA exhibited significantly higher c-Fos expression compared to the right TnA (left: 1755 ± 207 cells/mm^2^; right: 1341.4 ± 273 cells/mm^2^; Fig. 2f). No difference between the left and right hemisphere was found in the other analyzed brain regions for each group and sex (see Table S3 in the Supplementary Materials for all the paired *t*-test results).

### Behavioral results

When analyzing the behavioral parameter “distance moved” no main effect of “group” (*F*_(1, 28)_ = 1.554, *P* = 0.223), “sex” (*F*_(1, 28)_ = 0.019, *P* = 0.891), nor any interaction of “group x sex” (*F*_(1, 28)_ = 0.122, *P* = 0.730) was found. Similar results were obtained for the behavioral parameter “velocity.” Also in this case no effect of “group” (*F*_(1, 28)_ = 1.982, *P* = 0.170), “sex” (*F*_(1, 28)_ = 0.203, *P* = 0.656), nor any interaction of “group x sex” (*F*_(1, 28)_ = 0.208, *P* = 0.652) emerged.

Overall, chicks that were exposed to the biological motion stimulus moved to a similar amount and at a similar velocity (distance: 727 ± 125.7 cm; velocity: 0.92 ± 0.13 cm/s) compared to the ones exposed to the rigid motion stimulus (distance: 944.3 ± 114 cm; velocity: 1.20 ± 0.16 cm/s).

### General discussion

The results showed that different brain regions of the SBN (Newman 1999; O’Connell and Hofmann 2011; Goodson and Kingsbury 2013) show different responsiveness to the first exposure to biological motion in newly hatched visually naïve female chicks: the activity in the left preoptic area of the hypothalamus was higher after exposure to the biological motion stimulus, compared to nonbiological rigid motion stimulus. In contrast, activity in the left nucleus taeniae of the amygdala was higher in female chicks exposed to the rigid motion stimulus compared to the biological motion one.

Sex differences in response to biological motion have been reported previously in chicks. Miura and Matsushima (Miura and Matsushima 2012) reported that females on day 2 showed the biological motion preference only if first exposed to it for 2 h, while males expressed a preference for biological motion no matter which kind of motion they were first exposed to. It is possible that the difference in brain activity reported here is related to the higher social motivation exhibited from females in this polygynous species (Workman and Andrew 1989; Vallortigara and Andrew 1991; Vallortigara 1992). Indeed, Vallortigara et al. (Vallortigara et al. 1990) reported that females are more engaged in running for a social reinforcement than males. Recently, Zanon et al. reported a preference for point-light displays consisting of pairs of chicks facing each other (thus suggesting social interaction) in female but not in male chicks (Zanon et al. 2024).

Brain asymmetry in biological motion and in response to other cues to animacy have been also reported in chicks and in other species as well (Grossman et al. 2000; Lorenzi et al. 2017; Mayer et al. 2017a). Clearly the role of the left and right hemisphere may be different depending on the brain areas (and sex).

The preoptic area could be involved in discriminating biological motion at birth before any other visual stimulation. Previous experiments reported POA involvement during the first exposure to an alive conspecific moving but not when exposed to a taxidermized chick rigidly rotating on the vertical axis (Mayer et al. 2017a), while another study showed POA involvement during first exposure to motion animacy cues to self-propulsion (Lorenzi et al. 2017). Altogether, these results suggest a general involvement of POA at birth in discriminating the dynamic features of living creatures from inanimate ones.

The nucleus taeniae could instead be involved in discriminating the static features of living creatures. When chicks were first exposed to a taxidermized hen rotating on the vertical axis, TnA showed greater c-Fos expression than that of chicks exposed to the scrambled version of the same stimulus (Mayer et al. 2017b; Mayer et al. 2019). Although here we used point-light displays to rule out any role of static features of animate beings, the rigid rotating stimulus could still be recognized (by structure-from-motion mechanisms) as conveying the shape of a hen. Moreover, it is important to stress that TnA is largely involved in the fear responses (Cheng et al. 1999; Absil et al. 2002; Brito et al. 2011; Ikebuchi et al. 2012; Morandi-Raikova and Mayer 2020; Perez et al. 2020; Corrales Parada et al. 2021). Chicks exposed to the nonpreferred rigid motion stimulus could thus be more afraid than chicks exposed to the preferred biological motion stimulus. This could explain the greater c-Fos expression in this brain region and is in line with the general functions attributed to areas of the SBN, which regulates animals’ affective responses to social stimuli (Castagna et al. 1997; Balthazart and Ball 2007; Taziaux et al. 2008; Lischinsky and Lin 2020). It should be noted, nonetheless, that upregulation or downregulation immediate early genes expression may reflect the responses of different types of cells (e.g. excitatory and inhibitory neurons) which are present in that region, and therefore, it cannot be equated with the direction of a preference in behavioral response.

The present results did not find any involvement of the septum during exposure to biological motion. Previous experiments reported an involvement of septal nuclei during first exposure to an alive conspecific moving, and to self-propelled animate stimuli (Lorenzi et al. 2017; Mayer et al. 2017a, 2017b), it seems that biological motion mediated by point-light display involves different neural structures.

The main finding of our experiments is that recognizing and responding to biological motion recruits early and more primitive brain areas than those studied so far in humans and nonhuman species. Moreover, this selectivity is shown in completely visually naïve animals, in the absence of any specific visual experience. It could be argued that selectivity would emerge from a multisensory feedback as provided by movements of the embryo *in ovo*. However, this hypothesis has been recently discarded by evidence that pharmacological blocking of embryo movement has no effect on preferences for biological motion (Matsushima et al. 2022).

## Conclusion

In conclusion, moving from cortical/pallial to subcortical/subpallial regions seems necessary to gain a comprehensive understanding of the neural encoding of biological motion perception.

## Supplementary Material

S1_bhae262

S2_bhae262

SM_bhae262

## Data Availability

The dataset generated during the current study is available from the corresponding author on request.
