# Supplementary figures and images for: Noncortical coding of biological motion in newborn chicks’ brain
Source: Cereb Cortex. 2024 Jun 25;34(6):bhae262. doi: 10.1093/cercor/bhae262 (PMC11909798; doi:10.1093/cercor/bhae262)

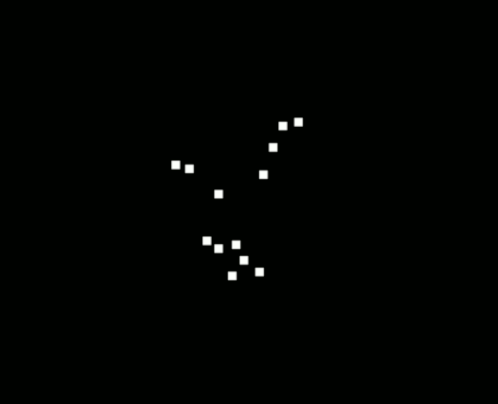

Supplement: S1_bhae262 [file s1_bhae262.gif]

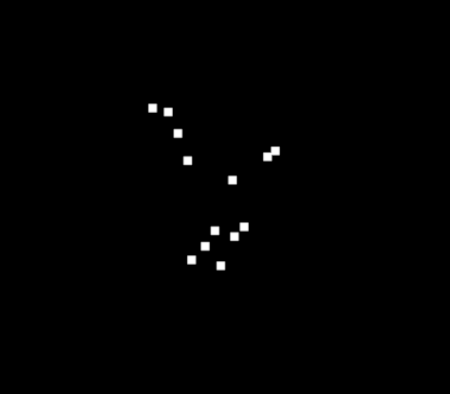

Supplement: S2_bhae262 [file s2_bhae262.gif]
