# Supplementary material for: Noncortical coding of biological motion in newborn chicks’ brain
Source: Cereb Cortex. 2024 Jun 25;34(6):bhae262. doi: 10.1093/cercor/bhae262 (PMC11909798; doi:10.1093/cercor/bhae262)
Supplement: SM_bhae262 [file sm_bhae262.docx]

## **Supplementary Material**

| **Main effect and interactions** | **Results** |
| --- | --- |
| area | **F_(1.890,47.246)_=20.614, p<0.001** |
| Area x group | **F_(1.890,47.246)_=4.679, p=0.015** |
| Area x sex | F_(1.890,47.246)_=0.714, p=0.487 |
| area x group x sex | F_(1.890,47.246)_=1.181, p=0.314 |
| hemisphere | F_(1,25)_=0.206, p=0.654 |
| hemisphere x group | F_(1,25)_=0.131, p=0.720 |
| hemisphere x sex | F_(1,25)_=0.003, p=0.955 |
| hemisphere x group x sex | F_(1,25)_=0.137, p=0.715 |
| area x hemisphere | **F_(2,50)_=4.955, p=0.011** |
| area x hemisphere x group | F_(2,50)_=0.166, p=0.847 |
| area x hemisphere x sex | F_(2,50)_=1.383, p=0.260 |
| area x hemisphere x group x sex | **F_(2,50)_=4.243, p=0.020** |
| group | F_(1,25)_=0.449, p=0.509 |
| sex | F_(1,25)_=0.002, p=0.967 |
| group x sex | F_(1,25)_=0.125, p=0.726 |

Table S1. Repeated measurements ANOVA. List of all interactions and effects.

| **Rigid motion stimulus - females** | **Left** | **Right** |
| --- | --- | --- |
| TnA | 1755 ± 207 | 1341.4 ± 273 |
| POA | 806.7 ± 138 | 108.3 ± 213 |
| Septum | 691.8 ± 169 | 750.2 ± 209 |
| **Rigid motion stimulus - males** | **Left** | **Right** |
| TnA | 1433.3±110 | 1280 ± 171 |
| POA | 996.7±175 | 1020 ± 189 |
| Septum | 591.6±176 | 842.9 ± 267 |
| **Biological motion stimulus - females** | **Left** | **Right** |
| TnA | 1186.3 ± 117 | 994.4 ± 180 |
| POA | 1352.4 ± 93 | 1440 ± 208 |
| Septum | 435 ± 274 | 782.2 ± 222 |
| **Biological motion stimulus - males** | **Left** | **Right** |
| TnA | 1095.3 ± 198 | 1081.7 ± 156 |
| POA | 923.3 ± 192 | 1266.7 ± 323 |
| Septum | 809.6 ± 131 | 644.5 ± 115 |

Table S2: Mean ± standard error of the mean of c-Fos-ir cells/mm^2^ for each group.

| **Rigid motion stimulus** | **females** | **males** |
| --- | --- | --- |
| TnA | **t_(7)_=2.551, p=0.038** | t_(7)_=0.859, p=0.419 |
| POA | t_(7)_=-1.209, p=0.266 | t_(7)_=-0.131, p=0.900 |
| Septum | t_(7)_=-0.264, p=0.799 | t_(7)_=-0.859, p=0.419 |
| **Biological motion stimulus** | **females** | **males** |
| TnA | t_(5)_=1.009, p=0.359 | t_(7)_=0.064, p=0.951 |
| POA | t_(6)_=-0.363, p=0.729 | t_(7)_=-1.382, p=0.209 |
| Septum | t_(6)_=-1.618, p=0.155 | t_(7)_=0.861, p=0.418 |

Table S3: Lateralization. *t*-tests between left and right hemispheres.

Video S1: The walking hen animation (biological motion stimulus).

Video S2: The rotating hen animation (non-biological motion stimulus).
